# Early Postoperative Outcomes with the Toumai^®^ Surgical System for Robot-Assisted Radical Prostatectomy: A Prospective Comparative Study with da Vinci^®^

**DOI:** 10.3390/cancers18091321

**Published:** 2026-04-22

**Authors:** Bernardo Rocco, Simona Presutti, Antonio Silvestri, Giuseppe Pallotta, Pierluigi Russo, Sara Mastrovito, Simone Assumma, Filippo Maria Turri, Enrico Panio, Francesco Rossi, Giovanni Battista Filomena, Filippo Gavi, Vincenzo Cavarra, Or Schubert, Giovanni Balocchi, Carlo Gandi, Francesco Pinto, Nazario Foschi, Angelo Totaro, Maria Chiara Sighinolfi

**Affiliations:** 1 Department of Urology, Fondazione Policlinico Universitario Agostino Gemelli IRCCS, 00168 Rome, Italyorshu1993@gmail.com (O.S.);; 2 Department of Medicine and Translational Surgery, Università Cattolica Del Sacro Cuore, 00168 Rome, Italy; 3 Department of Life Science, Health, and Health Professions, Unilink University, 00165 Rome, Italy; 4 Department of Obstetrics and Gynecology, Fondazione Policlinico Universitario Agostino Gemelli, 00168 Rome, Italy

**Keywords:** prostate cancer, robot-assisted radical prostatectomy, Toumai^®^ MT-1000, da Vinci^®^ Xi, robotic surgery, positive surgical margin, learning curve, CUSUM analysis

## Abstract

*Background*: Prostate cancer (PCa) imposes a substantial global health burden, with robot-assisted radical prostatectomy (RARP) established as the gold standard for localized disease. While da Vinci^®^ Xi maintains market dominance, Toumai^®^ MT-1000 offers a potentially cost-competitive alternative lacking prospective validation. *Objective*: To evaluate perioperative safety, oncologic quality (primary endpoint: positive surgical margins), early functional recovery (continence), and surgeon learning curve between Toumai^®^ MT-1000 (T-RARP) and da Vinci^®^ Xi RARP (DV-RARP) performed in high-volume European practice. *Materials and Methods*: This is a prospective single-center comparative study carried out at Policlinico Gemelli, Rome (May–November 2025), enrolling 80 patients with localized or locally advanced PCa, elected for radical prostatectomy and casually allocated to receive surgery with Toumai or the da Vinci robotic platform. The primary endpoint was the comparison of positive surgical margin (PSM) rates. Secondary endpoints included the comparison of operative time (skin-to-skin), estimated blood loss, length of hospital stay, 45-day postop outcomes, specifically Clavien–Dindo complications, urinary continence recovery (0–1 pad/day), and IIEF-5 scores. Learning curve was evaluated through the cumulative summation (CUSUM) analysis of operative times and linear regression of operative times (n = 80 cases). The analyses used STATA 19 with two-sided tests at *p* < 0.05 significance. Results: Baseline characteristics showed balance between cohorts (*p* > 0.05 for most covariates). Perioperative outcomes proved equivalent: median operative time (OT) was 192.5 min (IQR 165–230) for Toumai^®^ versus 183.5 min (IQR 147–225) for da Vinci^®^ Xi (*p* = 0.38); estimated blood loss (EBL) was 150 mL in both groups (*p* = 0.87); length of hospital stay (LOS) was 2 days in both groups (*p* = 0.92). PSM rates were identical at 17.5% (*p* = 0.79). Continence recovery reached 72.5% versus 80% (*p* = 0.43). Complications (Clavien–Dindo ≥ II) occurred in 7.5% versus 12.5% of cases (*p* = 0.45). The CUSUM analysis demonstrated operative time proficiency after only four procedures; operative time regression showed no significant trend (*p* = 0.38). *Conclusions*: Toumai^®^ MT-1000 demonstrates similar performance to da Vinci^®^ Xi across different RARP quality metrics, with no detectable learning curve for surgeons previously experienced with da Vinci. These findings support a safe integration of cost-effective platforms into clinical practice, pending multicenter randomized confirmation.

## Simple Summary

Prostate cancer represents a leading cause of morbidity among men, with robot-assisted radical prostatectomy established as the preferred surgical treatment for localized disease due to its enhanced precision and reduced complication rates. This prospective study conducted at a high-volume European center compares early postoperative outcomes of the novel Toumai MT-1000 robotic system against the established da Vinci Xi platform in 80 consecutive cases. The goal was to verify equivalence in surgery time, blood loss, hospital stay, continence recovery, and surgeon learning curves. The results demonstrate comparable performance across all metrics, with rapid proficiency achieved by experienced surgeons, suggesting that cost-effective alternatives can be safely integrated into clinical practice to broaden access to advanced surgical care.

## 1. Introduction

Prostate cancer (PCa) ranks as the second most common malignancy in men worldwide [1], with GLOBOCAN 2025 projections estimating 1.52 million new cases and 410,000 deaths [2,3]. In Europe, it accounts for ~330,000 annual diagnoses and ranks third among causes of cancer mortality in men, highlighting its profound clinical and economic impact across diverse healthcare systems [4,5,6]. Robot-assisted radical prostatectomy (RARP) has emerged as a cornerstone curative therapy for localized or locally advanced disease in patients with a life expectancy exceeding 10 years, offering superior perioperative outcomes—including reduced blood loss, shorter hospital stays [7,8,9], and faster functional recovery—compared to open or laparoscopic approaches [10,11,12,13]. da Vinci^®^ Xi (Intuitive Surgical, Sunnyvale, CA, USA) still commands the majority of the global market [14,15,16,17]; however, after the expiry of the patent, new robotic platforms [18] emerged, aiming to improve the cost-effectiveness of robotic surgery [19] and spread its worldwide adoption [20,21,22,23,24].

The Toumai^®^ MT-1000 (MicroPort MedBot, Shanghai, China; NMPA 2022, EU 2024) represents one of these next-generation robotic systems [25]. It displays a single boom design, an immersive console, and advanced features—four fully articulated arms (8.4 mm instruments, 7 DOF, 540° rotation), 3D-HD stereoscopic vision (1080 p/60 Hz, <50 ms latency), dual-console training, picture-in-picture display, one-click arm positioning, and haptic feedback (0.1 N/4 kHz/250 μs)—enabling precise pelvic dissection while minimizing trauma risks common in non-haptic platforms [26]. The Toumai^®^ MT-1000 also allows 5G-enabled telesurgery (≤70 ms delay over 2700 km) further promising to bridge global surgical expertise gaps [27,28].

Preliminary data from China (Chen et al. 2024 and Wei et al.) showed the feasibility of Toumai^®^ MT-1000 urological surgery, even when compared to da Vinci^®^ [29,30]. The rationale of the present study is to validate the clinical performance and safety outcomes of the Toumai^®^ MT-1000 robotic platform in a healthcare context different from that in which it was originally developed and implemented, thereby assessing the reproducibility and generalizability of previously reported results.

## 2. Material and Methods

### 2.1. Study Design

This is a single-center prospective study performed at the Policlinico Gemelli, Rome, a tertiary referral center for robotic surgery (>500 RARPs annually). The facility has been equipped with da Vinci^®^ Xi (Intuitive Surgical, Sunnyvale, CA, USA), Toumai^®^ MT-1000 (MicroPort MedBot, Shanghai, China), Hugo^TM^RAS (Medtronic, Minneapolis, MN, USA), and Versius^®^ (CMR Surgical, Cambridge, UK), the latter up to 2024. Between May and November 2025, we enrolled 80 consecutive patients with clinically localized or locally advanced prostate cancer (cT1-cT2c N0 M0) undergoing RARP as the primary treatment. The patients were allocated 1:1 to either da Vinci^®^ Xi (Group A, n = 40) or Toumai^®^ MT-1000 (Group B, n = 40) based on surgical scheduling availability. All procedures were performed by eight surgeons experienced in da Vinci^®^ RARP (each with ≥100 prior cases), minimizing learning curve bias and isolating true platform-specific performance differences.

Data from 40 consecutive patients per arm (n = 80 total) were collected, including baseline demographics, preoperative multiparametric MRI (mpMRI) reports, biopsy pathology, intraoperative logs, final pathology, and protocolized 45-day postoperative assessments.

Written informed consent was obtained from all patients for institutional RARP database inclusion and specific URO-PROGRESS registry participation. The patient data were fully anonymized per GDPR 2016/679 and AIFA observational study regulations, following the CONSORT 2010 (non-pharmacological trials) and STROBE (observational studies) guidelines.

### 2.2. Inclusion and Exclusion Criteria

Eligible patients were men aged 40–75 years old with biopsy-confirmed prostate cancer (ISUP grade group 1–5, PSA ≤ 40 ng/mL), complete preoperative staging via mpMRI fusion biopsy plus PSMA-PET or CT and bone scintigraphy, and suitability for elective RARP without neoadjuvant therapy. A minimum 45-day follow-up with complete primary endpoint data was required.

We excluded patients for prior major pelvic surgery or prostate radiotherapy, neoadjuvant hormonal/chemoradiotherapy, ASA score > 3, BMI > 35 kg/m^2^, >20% missing key data, or withdrawal/lost to follow-up before 45 days.

### 2.3. Surgical Technique

RARP was performed using a transperitoneal approach, with identical surgical steps across both the Toumai^®^ MT-1000 and da Vinci^®^ Xi platforms to rigorously eliminate technique-related confounding and precisely isolate inherent system performance differences. Surgeons selected anterior (Retzius) or posterior (Montsouris) approaches according to their standard surgical practice.

Standard W-shaped trocar configuration was maintained across platforms: supraumbilical optical access via open technique (8 mm da Vinci^®^ Xi, 10 mm Toumai^®^ MT-1000), three additional 8 mm robotic trocars along a transverse line 14 cm from the pubic symphysis, 10 mm Air-Seal assistant trocar in the right flank, and 5 mm auxiliary trocar in the right hypochondrium (12–15 mmHg pneumoperitoneum).

The core surgical technique remained identical across systems, employing the same instrumentation portfolio—featuring monopolar scissors, Maryland bipolar forceps or fenestrated grasper, and needle driver—together with Stratafix 3-0 barbed sutures (Ethicon, Somerville, NJ, USA), Hem-o-lok clips (Teleflex, Wayne, PA, USA), and hemostatic agents like Floseal (Baxter, Deerfield, IL, USA) or Tabotamp (Ethicon, Somerville, NJ, USA). The surgeons worked with either 0° or 30° optics based on individual preference, following a consistent sequence that began with prostatic defatting, progressed through bilateral endopelvic fascia incision and pubo-prostatic ligament division, bladder neck dissection to the seminal vesicle plane, vascular pedicle control, postero-lateral dissection with nerve sparing, apex management via cold-knife urethrotomy, en bloc prostatovesiculectomy, Rocco posterior urethral reconstruction, and continuous van Velthoven vesicourethral anastomosis over a 16 Ch Foley catheter. Extended pelvic lymph node dissection (bilateral external iliac and obturator fossa) was routinely performed according to nomogram-based risk assessment. A 19 Ch spiral drain was selectively placed in the pelvis (exiting the left lateral port) guided by intraoperative findings such as estimated blood loss exceeding 300 mL, extent of lymphadenectomy, or concerns regarding anastomotic integrity, followed by multilayer abdominal closure and Vicryl Rapide 4-0 skin suturing (Ethicon, Somerville, NJ, USA).

### 2.4. Endpoints

The primary endpoint was the positive surgical margin (PSM) rate, defined as the presence of cancer cells in direct contact with inked specimen surfaces on final pathology examination.

Secondary endpoints encompassed a comprehensive assessment of perioperative performance—such as operative time (OT, skin-to-skin) and estimated blood loss (EBL)—as well as postoperative recovery metrics including length of hospital stay (LOS), 45-day Clavien–Dindo complications, undetectable PSA nadir (<0.1 ng/mL in accordance with EAU guidelines), urinary continence recovery (0–1 pad/day at 45 days), and early erectile function via the IIEF-5 scores.

### 2.5. Statistical Analysis

Baseline demographics and perioperative characteristics were summarized using descriptive methods. The Shapiro–Wilk test was used to examine whether variables followed a normal distribution. Categorical variables were reported as percentages, whereas continuous variables were reported as mean or median values along with interquartile ranges (IQR). Adjustment variables included age at surgery, baseline questionnaire scores, preoperative serum PSA levels, and prostate volume. All tests were two-sided, with statistical significance at *p* < 0.05. The analyses were performed using STATA 19 (StataCorp LLC, College Station, TX, USA). The learning curve for operative time of the surgeon that reached a sufficient number of cases was evaluated using the cumulative summation (CUSUM) analysis. CUSUM is commonly used to plot data from successive procedures, transforming individual measurements into a running total of differences relative to the group mean. In the literature, the inflection point of the curve is commonly interpreted as the transition from the initial learning phase to a proficiency phase. This method typically produces a bell-shaped curve, in which the initial upward phase reflects procedures exceeding the mean operative time, followed by a downward phase representing procedures performed below the mean.

Although not without limitations, CUSUM provides a clear visual estimate of the number of procedures required to achieve proficiency, indicated by a progressively decreasing curve or stabilization around zero.

Operative times (skin-to-skin) for the first 80 institutional procedures were further analyzed using simple linear regression for both platforms, modeling operative time as a function of sequential case number (1–80), independent of the surgeon. Model adequacy was assessed using the coefficient of determination (R^2^), which was low (R^2^ = 0.011), indicating that sequential case number explained only a negligible proportion of the variability in operative time.

Residual diagnostics were evaluated through visual inspection of residual plots, which did not reveal any major deviations from model assumptions (linearity and homoscedasticity). The following STATA syntax was adopted to run the tests: swilk, ttest, tabstat, ranksum, tabulate, chi2, column, mixed, and margins.

## 3. Results

### 3.1. Baseline Characteristics

From May to November 2025, 80 patients met inclusion criteria and completed 45-day follow-up. Table 1 reports a descriptive analysis of all clinical preoperative variables in the whole cohort. Baseline characteristics were balanced between groups (Table 2), with some statistically significant—yet clinically minor—differences reflecting real-world practice variations. Specifically, the Toumai^®^ MT-1000 group showed higher rates of diabetes (17.5% vs. 2.5%, *p* = 0.025), whereas the da Vinci^®^ Xi group had greater prevalence of coronary heart disease (17.5% vs. 2.5%, *p* = 0.025). A different ASA distribution (*p* = 0.034) was evident, with more ASA1 and ASA3 patients, increased preoperative BPH therapy (22.5% vs. 5%, *p* = 0.023) and DE therapy (10% vs. 0%, *p* = 0.04), plus prior active surveillance (10% vs. 0%, *p* = 0.04) in the da Vinci group^®^. More advanced clinical staging with palpable disease was more common (cT2 in 25% vs. 5%, *p* = 0.02) in the Toumai^®^ MT-1000 group, as well as a trend toward higher PI-RADS scores.

### 3.2. Pathological Findings

As far as the primary endpoint is concerned, the rate of PSM was identical and it was 17.5% (7/40) for both groups (Table 3). Median PSM length was 5 mm (IQR 4–7) and 4 mm (IQR 2–10) for Toumai^®^ MT-1000 and da Vinci^®^ Xi cases, respectively (*p* = 0.79) (Table 3). Pathological staging showed balanced distributions between the Toumai^®^ MT-1000 and da Vinci^®^ Xi groups, (*p* = 0.18); however, pT3b was higher in the Toumai^®^ MT-1000 group (17.5% vs. 2.5%), consistent with the preoperative finding of a greater amount of palpable disease. Despite this adverse distribution, PSM rates remained equivalent (17.5% both cohorts, *p* = 0.79) with comparable PSM lengths (median 4 mm vs. 5 mm, *p* = 0.75).

### 3.3. Intra-, Peri-, and Postoperative Outcomes

Intra-, perioperative, and postoperative outcomes are reported in Table 3.

Operative time (skin-to-skin) was similar between platforms, specifically 192.5 min (IQR 165–230) for Toumai^®^ MT-1000 and 183.5 min (IQR 147–225) for da Vinci^®^ Xi, yielding a clinically negligible median difference of 9 min (*p* = 0.38). EBL was 150 mL (IQR 100–200) in both cohorts (*p* = 0.87). No intraoperative complications, conversions to open surgery, device malfunctions, need to modify surgical strategy due to platform limitations, grade ≥ IV events, nor device-related adverse events occurred in either cohort. Median length of stay was 2 days (IQR 2–3) for both groups (*p* = 0.92) and time to catheter removal was similar between groups as well (9 days, IQR 7–10 and 7–11 for Toumai^®^ MT-1000 and da Vinci^®^ Xi, respectively; *p* = 0.56). Postoperative complications (Clavien–Dindo ≥ II at 30 days) occurred in 3/40 (7.5%) Toumai^®^ MT-1000 cases versus 5/40 (12.5%) da Vinci^®^ Xi cases (*p* = 0.45). Specifically, the Toumai^®^ MT-1000 group recorded four events—fever treated with antibiotics (grade II), vesicourethral anastomotic leakage (grade II), anastomotic stenosis scheduled for urethrotomy (grade IIIb), and ureteral leak managed conservatively with ureteral stenting (grade IIIa)—while the da Vinci^®^ Xi group experienced five events—bleeding requiring surgical intervention (grade IIIb), bleeding managed with blood transfusion (grade II), and three fever cases treated with antibiotics (grade II). Tumor volume was higher in Toumai (2 vs. 1.16 cc, *p* = 0.05) and adjusted statistical analysis showed no PSM association.

Postoperative functional outcomes were comparable between groups. Continence recovery (0–1 pads/day) at 1 month was achieved in 80% (32/40) versus 72.5% (29/40) patients (*p* = 0.43) (Table 4).

### 3.4. Cumulative Summation (CUSUM) and Scatterplot

**Figure 1:**
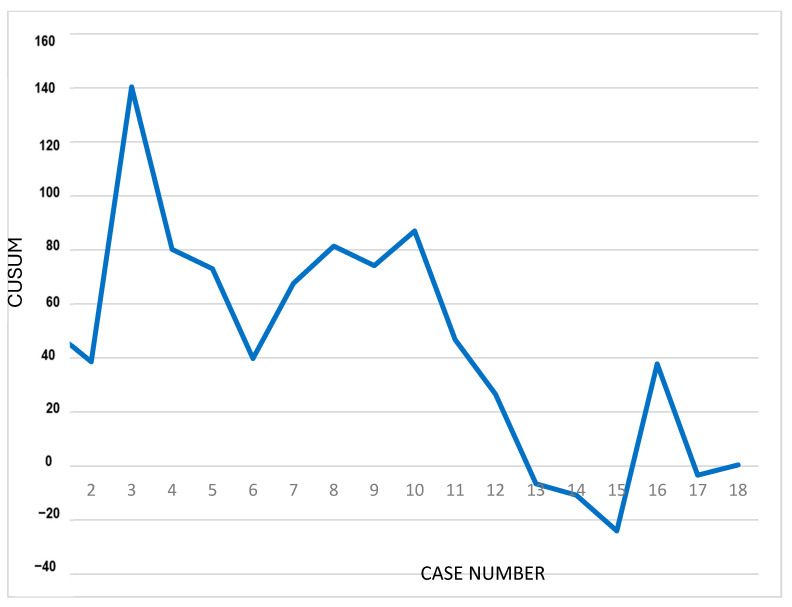
Cumulative summation (CUSUM) analysis for evaluating performance trends with the Toumai^®^ MT-1000 platform in terms of operative time.

The cumulative summation (CUSUM) analysis was performed to evaluate performance trends with the Toumai^®^ MT-1000 platform in terms of operative time. Only one surgeon achieved an adequate case volume to allow for a reliable assessment; therefore, a single CUSUM curve was generated. The curve showed an early peak of the bell-shaped pattern at the third procedure out of a total of 17 cases, followed by a rapid stabilization phase, suggesting early procedural consistency (Figure 1).

In parallel, the linear regression analysis demonstrated no significant association between operative time and case sequence (β = −0.23, *p* = 0.34), with a very low explanatory power (R^2^ = 0.011), confirming the absence of a meaningful learning trend. When analyzed separately by platform, linear regression showed no significant association between operative time and case sequence for either system. The da Vinci^®^ platform demonstrated no trend (β = 0.09, *p* = 0.91; R^2^ = 0.0004), while the Toumai^®^ MT-1000 platform showed a non-significant decreasing trend (β = −0.81, *p* = 0.21; R^2^ = 0.041), indicating minimal explanatory power. These findings confirm the absence of a meaningful reduction in operative time over consecutive cases.

**Figure 2:**
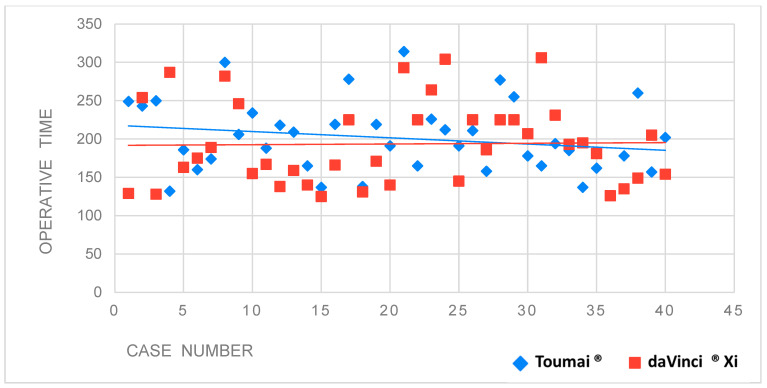
Scatterplot showing absence of linear correlation between OT and procedural sequence, with no reduction in OT over time.

Mean operative times remained comparable between the Toumai^®^ MT-1000 and da Vinci^®^ Xi groups despite the longer historical use of the latter (Figure 2).

## 4. Discussion

To the best of our knowledge, this is the wider series comparing the newly introduced Toumai^®^ MT-1000 with the well-established da Vinci^®^ Xi platform for radical prostatectomy and pelvic nodal dissection; furthermore, we initially provided insights into the learning curve and the transition to the Toumai^®^ MT-1000 platform for surgeons previously trained on da Vinci^®^ Xi.

Our outcomes suggest that the performance of Toumai^®^ MT-1000 may be similar to that of da Vinci^®^ Xi from its very early introduction across key perioperative parameters, including surgical margins, operative time, estimated blood loss, length of hospital stay, and early postoperative complications. The finding that higher-risk cases were preferentially assigned to the Toumai platform by scheduling, yet demonstrated equivalent PSM rates, supports the robustness of its oncologic performance under less favorable baseline conditions. These findings are consistent with previous evidence from earlier studies performed in China, where Toumai^®^ MT-1000 demonstrated adequate safety and feasibility in smaller RARP series (Tan, Chen). Notably, the present study represents the first structured evaluation of this platform in a European clinical setting, thereby assessing the reproducibility of Toumai^®^ MT-1000 RARP in a healthcare environment different from that in which the system was originally developed. This context may involve variations in patient baseline characteristics, disease presentation, and surgical workflows.

Chen et al. recently conducted a randomized prospective comparative study demonstrating overlapping oncological outcomes between the Toumai^®^ MT-1000 system and the da Vinci^®^ Xi for both RARP and robot-assisted partial nephrectomy (RAPN). Specifically for RARP, no significant differences emerged in terms of PSM rates, blood loss, hospital stay, and postoperative complications, thereby confirming equivalence in our primary endpoints (PSM) and secondary endpoints (continence recovery, undetectable PSA at 45 days). In the series from Chen, operative time was longer for Toumai^®^ MT-1000 compared to the da Vinci^®^ Xi (138 vs. 108 min); in the current series, similar OTs were found between systems, overall, seemingly longer than those reported by Chen, likely due to the inclusion of cases with pelvic nodal dissection.

Remarkably, we did not experience severe malfunctions nor instrument failures that could have posed intraoperative risk during procedural steps close to vessels like pelvic lymphadenectomy. In our series, node yield was similar between robotic systems, thus highlighting the efficacy and safety of Toumai^®^ MT-1000 also for complex procedures/anatomical sites. Once more, the safety of Toumai^®^ MT-1000 has been confirmed also by Chen, who reported comparable perioperative metrics for partial nephrectomy, including blood loss and complication rates.

Tan et al. recently published a preliminary prospective single-center analysis of 20 urological procedures—including five RAPNs; the authors reported satisfactory outcomes with no need for conversions (100% success rate) and acceptable perioperative outcomes (i.e., hemoglobin drop of 1.2 g/dL, mean hospital stay of 9.4 days, minor complications Clavien–Dindo I/II at 40% for RAPN). However, they highlighted long operative time with Toumai^®^ MT-1000; this finding was attributed to the adaptation period caused by surgical team’s unfamiliarity with the new features of the robot.

In contrast, we previously described an easy transition to Toumai^®^ MT-1000 from da Vinci^®^ users in a preclinical series on animal models; experienced surgeons with over 200 prior RARP cases demonstrated immediate proficiency without formal retraining, as evidenced by comparable operative times, precise instrument control, and ergonomic comfort from the first procedure, further reinforcing the platform’s intuitive design, haptic feedback similarity, and seamless accessibility for transitioning operators [31].

In fact, a central and novel aspect of the current study is the learning curve analysis. One of the major barriers to the adoption of new robotic platforms is the perceived need for extensive retraining, particularly for surgeons already proficient with the da Vinci^®^ system. The CUSUM findings indicate that an experienced robotic surgeon may transition to the Toumai^®^ MT-1000 platform without undergoing a prolonged, platform-specific learning curve. The very early inflection point observed after only three procedures suggests rapid adaptation rather than a true learning phase, suggesting skill transferability between robotic systems. The absence of a decreasing operative time trend on linear regression analysis reinforces this interpretation, as neither platform demonstrated progressive reduction in time over consecutive procedures. Similar mean operative times between cohorts show that technological differences between platforms may have minimal impact on operative performance when procedures are performed by surgeons already proficient in robotic surgery. Collectively, these results suggest that prior robotic experience, rather than specific platform training, may be the primary determinant of efficiency.

If confirmed in larger cohorts, these results could have significant implications for health systems, as they would reduce the costs, delays, and logistical challenges associated with platform transition and formal certification pathways.

The clinical relevance of these findings extends beyond individual surgical outcomes. The availability of alternative robotic platforms could promote competition, reduce procedural costs, and improve access to minimally invasive surgery. Moreover, features such as haptic feedback and 5G-enabled telesurgery may offer additional advantages in training, mentoring, and equitable distribution of surgical expertise, although these aspects were not directly evaluated in the present study.

The clinical equivalence between Toumai MT-1000 and da Vinci Xi demonstrated here has important economic implications amid rising healthcare costs. Despite technological superiority, da Vinci Xi entails acquisition costs (>$2.5M), annual maintenance (~$150–200K), and proprietary consumables ($1500–3000/procedure), limiting access to high-resource centers [20].

Patent expirations have spurred next-generation platforms—including Toumai^®^, Hugo^TM^RAS, Versius^TM^, and others—with €1–1.5M acquisition costs and 20–50% lower consumables via modular designs and standardized components. Future studies incorporating detailed cost-effectiveness analyses (e.g., incremental cost per quality-adjusted life year gained) will be essential for guiding healthcare policy decisions [19,20,21].

Several limitations must be acknowledged. First, the non-randomized design may have introduced selection bias despite balanced baseline characteristics. However, it should be remarked that the allocation was casual: the higher clinical stage in the Toumai^®^ MT-1000 cohort together with the higher eligibility to nodal dissection confirm this argument. Second, the limited sample size and short follow-up preclude conclusions regarding long-term oncologic and functional outcomes. Third, all procedures were performed by experienced multiplatform surgeons at a high-volume center. While CUSUM demonstrated rapid platform proficiency transfer (3–4 cases), these results cannot be extrapolated to robotic-naïve or low-volume centers where PSM and complication rates are predictably higher. Dedicated studies in low-volume settings are essential. Finally, economic outcomes, including cost-effectiveness and resource utilization, were not formally analyzed and warrant dedicated investigation.

Despite these limitations, the study is the first clinical comparative experience with Toumai^®^ MT-1000 performed outside China. Up to the present moment, it also represents the first attempt to analyze skill transference between the da Vinci^®^ Xi and Toumai^®^ MT-1000 systems.

## 5. Conclusions

This preliminary single-center European study suggests that robot-assisted laparoscopic radical prostatectomy performed with the Toumai^®^ MT-1000 platform yields outcomes comparable to the da Vinci^®^ Xi system in terms of perioperative safety, early oncologic quality, and short-term functional recovery when procedures are carried out by experienced robotic surgeons.

Furthermore, the absence of a detectable learning curve in high-volume operators, as demonstrated by the CUSUM analysis, indicates that platform transition may be feasible without compromising surgical performance or requiring prolonged retraining.

While these findings must be interpreted with caution due to the exploratory nature of the data and limited follow-up, they provide an important proof of concept supporting the safe integration of next-generation robotic systems into European clinical practice. Larger prospective studies with longer follow-up and formal health–economic evaluations are required to confirm these results and to define the role of emerging robotic platforms in the evolving landscape of prostate cancer surgery.

## Figures and Tables

**Table 1:** Descriptive analysis.

Age, Years (Mean ± SD)	66.6 ± 6.71
BMI, kg/m^2^ (mean ± SD)	25.6 ± 3.01
Diabetes, n (%)	8 (10)
ASA, n (%)	
1	4 (5)
2	57 (71.25)
3	19 (23.75)
Hypertension, n (%)	36 (45)
Coronary heart disease, n (%)	8 (10)
CCI, n (%)	
2	1 (1.25)
3	11 (13.75)
4	29 (36.25)
5	21 (26.25)
6	14 (17.5)
7	2 (2.5)
9	1 (1.25)
10	1 (1.25)
Preoperative PSA, ng/mL, median (IQR)	7.1 (5.07–10)
Prior active surveillance, n (%)	4 (5)
Prostate volume, cc, median (IQR)	41 (32.5–62)
Gleason grade group, n (%)	
ISUP 1	21 (26.25)
ISUP 2	28 (35)
ISUP 3	15 (18.75)
ISUP 4	15 (18.75)
ISUP 5	1 (1.25)

BMI = body mass index; SD = standard deviation; CCI = Charlson Comorbidity Index; IQR = interquartile range; ISUP = International Society of Urological Pathology.

**Table 2:** Baseline characteristics.

Variables	da Vinci^®^ Xi (n = 40)	Toumai^®^ MT-1000 (n = 40)	*p* Value
Age, years (mean ± SD)	66.5 (±7.38)	66.8 (±6.06)	0.58
BMI, kg/m^2^ (mean ± SD)	25.75 ± 3.31	25.47 ± 2.71	0.34
Diabetes, n (%)	1 (2.5%)	7 (17.5%)	0.025
Hypertension, n (%)	18 (45%)	18 (45%)	1
Coronary heart disease, n (%)	7 (17.5%)	1 (2.5%)	0.025
ASA, n (%)			0.034
1	4 (10)	0 (0)	
2	24 (60)	33 (82.5)	
3	12 (30)	7 (17.5)	
CCI, n (%)			0.57
2	1 (2.5)	0 (0)	
3	6 (15)	5 (12.5)	
4	13 (32.5)	16 (40)	
5	10 (25)	11 (27.5)	
6	6 (15)	8 (20)	
7	2 (5)	0 (0)	
9	1 (2.5)	0 (0)	
10	1 (2.5)	0 (0)	
Prior BPH endoscopic surgery, n (%)	3 (7.5)	1 (2.5)	0.30
BPH therapy, n (%)	9 (22.5)	2 (5)	0.023
Preoperative DE therapy, n (%)	4 (10)	0 (0)	0.04
Previous abdominal surgery, n (%)	18 (45)	17 (42.5)	0.82
Preoperative PSA, ng/mL, median (IQR)	6.44 (5.02–9.8)	7.3 (5.25–10.7)	0.29
Clinical stage, CT, n (%)			0.02
cT1c	38 (95)	30 (75)	
cT2a	2 (5)	9 (22.5)	
cT2b	0 (0)	1 (2.5)	
PI-RADS score, n (%)			0.14
3	8 (20)	4 (10)	
4	25 (62.5)	22 (55)	
5	7 (17.5)	14 (35)	
Prostate volume, cc, median (IQR)	41 (30–57)	42 (37–67)	0.41
Gleason grade group, n (%)			0.46
ISUP 1	12 (30)	8 (20)	
ISUP 2	15 (37.5)	14 (35)	
ISUP 3	8 (20)	7 (17.5)	
ISUP 4	5 (12.5)	10 (25)	
ISUP 5	0 (0)	1 (2.5)	
Prior active surveillance, n (%)	4 (10)	0 (0)	0.04
Clinical N stage, n (%)	2 (5)	4 (10)	0.39
Imaging modality used for systemic staging (if not in the list, specify)			0.07
Not performed, n (%)	21 (52.5)	12 (30)	
PET-PSMA, n (%)	2 (5)	2 (5)	
Pet-choline, n (%)	12 (30)	15 (37.5)	
CT alone, n (%)	0 (0)	3 (7.5)	
Bone scan, n (%)	4 (10)	2 (5)	
CT + bone scan, n (%)	1 (2.5)	6 (15)	
IPSS score, n (%)			0.22
Moderate (8–19)	18 (45)	15 (37.5)	
Mild (1–7)	21 (52.5)	20 (50)	
Severe (20–35)	1 (2.5)	5 (12.5)	
IIEF-5 score, n (%)			0.92
Normal 22–25	17 (42.5)	14 (35)	
Mild 17–21	7 (17.5)	6 (15)	
Mild-moderate 12–16	5 (12.5)	7 (17.5)	
Moderate 8–11	3 (7.5)	3 (7.5)	
Severe 5–7	8 (20)	10 (25)	

BMI = body mass index; SD = standard deviation; CCI = Charlson Comorbidity Index; BPH = benign prostatic hyperplasia; IQR = interquartile range; ISUP = International Society of Urological Pathology.

**Table 3:** Intra-, peri-, and postoperative outcomes.

Variables	da Vinci^®^ Xi (n = 40)	Toumai^®^ MT-1000 (n = 40)	*p* Value
OT, min, median (IQR)	183.5 (147–225)	192.5 (165–230)	0.38
LOS, days, median (IQR)	2 (2–3)	2 (2–3)	0.92
Catheterization time, days, median (IQR)	9 (7–11)	9 (7–10)	0.56
Primary Gleason at final pathology, n (%)			0.14
3	31 (77.5%)	25 (62.5%)	
4	9 (22.5%)	15 (37.5%)	
5	0 (0%)	0 (0%)	
Secondary Gleason at final pathology, n (%)			0.37
3	16 (40%)	14 (35%)	
4	23 (57.5%)	22 (55%)	
5	1 (2.5%)	4 (10%)	
pT stage, n (%)			0.18
pT2a	2 (5%)	4 (10%)	
pT2b	1 (2.5%)	1 (2.5%)	
pT2c	30 (75%)	23 (57.5%)	
pT3a	6 (15%)	5 (12.5%)	
pT3b	1 (2.5%)	7 (17.5%)	
pT4	0 (0%)	0 (0%)	
pN stage, n (%)			0.13
N0	4 (10%)	11 (27.5%)	
N1	1 (2.5%)	1 (2.5%)	
Nx	35 (87.5%)	28 (70%)	
Number of lymph nodes dissected, median (IQR)	17 (12–25)	12 (5–21)	0.33
Tumor volume at final pathology, median (IQR)	1.16 (0.6–2.7)	2 (1.07–3.4)	0.05
PSM length, mm, median (IQR)	4 (2–10)	5(4–7)	0.75
PSM, n (%)			1
Negative	33 (82.5)	33(82.5)	
Positive	7 (17.5)	7 (17.5)	
Postoperative complication, n (%)	5 (12.5)	3 (7.5)	0.45
Clavien–Dindo grade, n (%)			0.58
I	0 (0)	0 (0)	
II	4 (10)	2 (5)	
III a	0 (0)	1 (2.5)	
III b	1 (2.5)	1 (2.5)	
IV a	0 (0)	0 (0)	
IV b	0 (0)	0 (0)	
V	0 (0)	0 (0)	

PSM = positive surgical margin; OT = operative time; LOS = length of hospital stay.

**Table 4:** Postoperative functional outcomes.

Variables	da Vinci^®^ Xi (n = 40)	Toumai^®^ MT-1000 (n = 40)	*p* Value
Detectable PSA at 1 month, n (%)	3 (7.5%)	5 (12.5%)	0.45
Continence at 1 month, 0–1 pads, n (%)	32 (80%)	29 (72.5%)	0.43

## Data Availability

No new data were created or analyzed in this study. Data sharing is not applicable to this article.
